# Parasite motility is critical for virulence of African trypanosomes

**DOI:** 10.1038/s41598-018-27228-0

**Published:** 2018-06-14

**Authors:** Michelle M. Shimogawa, Sunayan S. Ray, Neville Kisalu, Yibo Zhang, Quanjie Geng, Aydogan Ozcan, Kent L. Hill

**Affiliations:** 1 Department of Microbiology, Immunology and Molecular Genetics, University of California, Los Angeles, CA 90095 USA; 2 Electrical and Computer Engineering Department, University of California, Los Angeles, CA 90095 USA; 3 Bioengineering Department, University of California, Los Angeles, CA 90095 USA; 4 California NanoSystems Institute (CNSI), University of California, Los Angeles, CA 90095 USA; 5 Molecular Biology Institute, University of California, Los Angeles, CA 90095 USA; 6 Present Address: Department of Ophthalmology, University of California, San Diego, La Jolla, CA 92093 USA; 7 Present Address: Cellular Immunology Section, Vaccine Research Center, National Institute of Allergy and Infectious Diseases, National Institutes of Health, Bethesda, MD 20892 USA

## Abstract

African trypanosomes, *Trypanosoma brucei* spp., are lethal pathogens that cause substantial human suffering and limit economic development in some of the world’s most impoverished regions. The name *Trypanosoma* (“auger cell”) derives from the parasite’s distinctive motility, which is driven by a single flagellum. However, despite decades of study, a requirement for trypanosome motility in mammalian host infection has not been established. LC1 is a conserved dynein subunit required for flagellar motility. Prior studies with a conditional RNAi-based LC1 mutant, RNAi-K/R, revealed that parasites with defective motility could infect mice. However, RNAi-K/R retained residual expression of wild-type LC1 and residual motility, thus precluding definitive interpretation. To overcome these limitations, here we generate constitutive mutants in which both LC1 alleles are replaced with mutant versions. These double knock-in mutants show reduced motility compared to RNAi-K/R and are viable in culture, but are unable to maintain bloodstream infection in mice. The virulence defect is independent of infection route but dependent on an intact host immune system. By comparing different mutants, we also reveal a critical dependence on the LC1 N-terminus for motility and virulence. Our findings demonstrate that trypanosome motility is critical for establishment and maintenance of bloodstream infection, implicating dynein-dependent flagellar motility as a potential drug target.

## Introduction

Flagellated protozoa include many human pathogens of medical and economic importance, such as trypanosomatids, trichomonads, *Giardia*, and *Plasmodium* parasites^1–3^, which together have a devastating impact on global public health^4–7^. These pathogens employ flagella for driving cell propulsion yet the role of parasite motility in pathogenesis remains an unanswered question. Flagellum motility is driven by thousands of dynein molecular motors arrayed along doublet microtubules of the axoneme^8,9^. Loss of function studies in *Trypanosoma brucei* confirmed the requirement for dyneins in motility of these organisms and implicated motility in transmission through the tsetse fly vector^10–12^. A role for motility during infection of a mammalian host however, has been controversial, owing in part to the fact that loss of dyneins or other axonemal proteins is lethal in the mammalian-infectious life cycle stage, even when the parasites are grown in culture^11,13–16^. To overcome this, Ralston and colleagues^16^ used an inducible system to express a mutant copy of the outer arm dynein subunit LC1, LC1-K203A/R210A, while simultaneously knocking down the wild type (WT) gene with RNAi. This approach disrupted dynein function but kept the dynein motor intact, resulting in a motility mutant that was viable in culture. Surprisingly, the resulting mutant, hereafter referred to as RNAi-K/R, remained virulent in mice^17^, indicating trypanosomes with defective motility can still infect a mammalian host. This result led to the conclusion that normal parasite motility is not required for virulence. It was noted however, that the RNAi-K/R mutant does retain some propulsive motility, particularly in high viscosity environments such as blood^17^, leaving open the possibility that virulence is due to this residual propulsive motility.

RNAi does not completely block gene expression in the RNAi-K/R mutant^16^ and residual WT LC1 expression might therefore be responsible for the remaining propulsive motility and virulence of these mutants. The *T. brucei* axoneme is roughly 25 μm long with nine axonemal microtubule doublets, each containing four outer arm dyneins per 96-nm repeating unit^18^. Each outer arm dynein contains one LC1 subunit^19,20^ and the axoneme therefore contains an estimated 9000 copies of LC1. Therefore, a mixture of WT and mutant LC1 is likely incorporated into the axoneme in the RNAi-K/R mutant and this may provide sufficient motility to sustain viability in culture and pathogenesis in mice. To address this possibility, we generated a constitutive mutant in which both LC1 alleles are replaced with the LC1-K203A/R210A mutant transgene^16^, so that no WT LC1 is available. Motility of the resulting double knock-in (DKI) mutant is substantially reduced compared to that of the RNAi-K/R mutant, indicating residual expression of WT LC1 is indeed responsible for residual motility observed in the latter. Importantly, we find that the DKI mutant is essentially devoid of propulsive motility and is unable to mount a bloodstream infection in mice. Our combined results demonstrate parasite motility is a virulence factor and provide insight into potential roles for motility during infection.

## Results

### Constitutive LC1 mutant provides a complete block of propulsive motility

**Figure 1:**
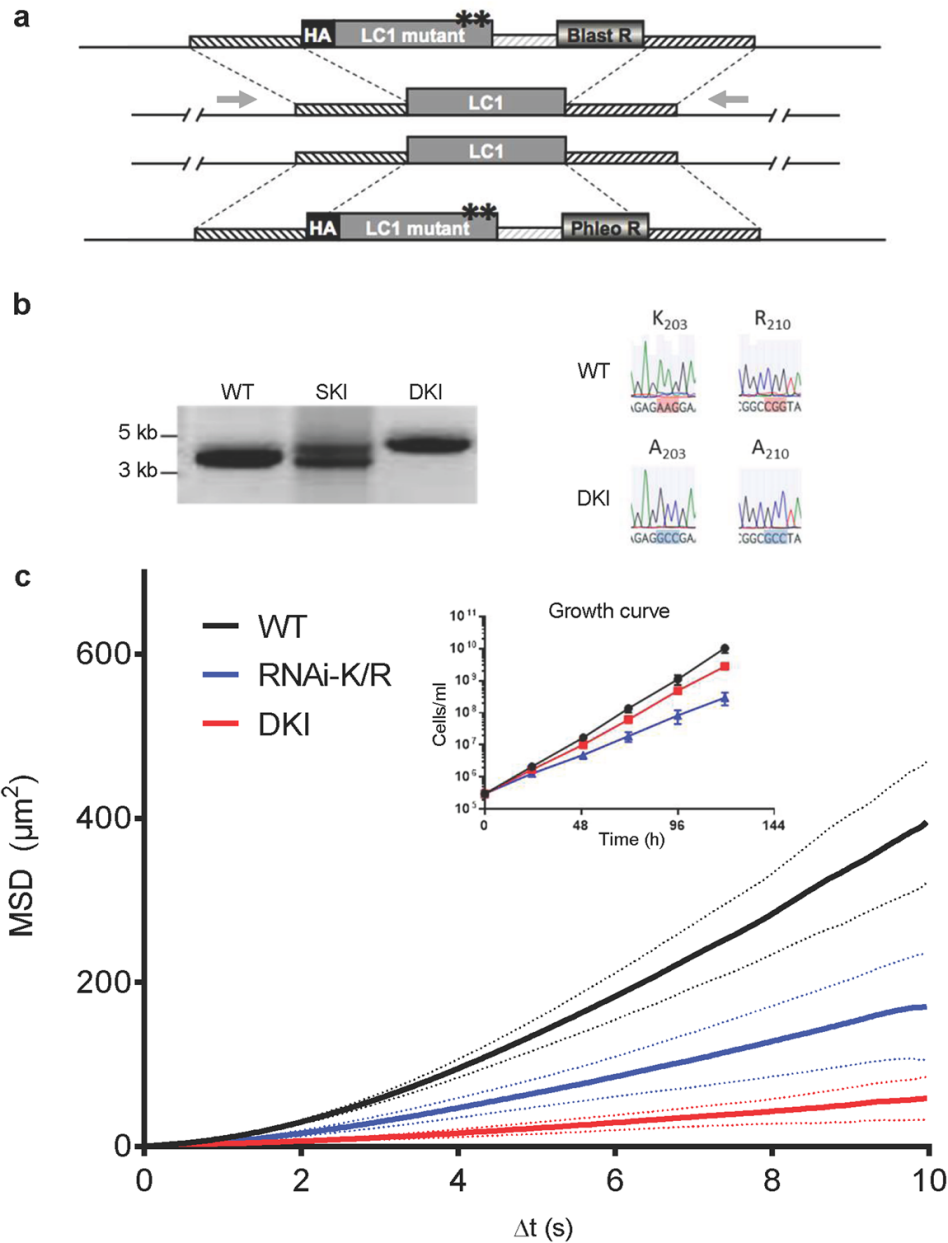
Constitutive LC1 double knock-in (DKI) motility mutant. (**a**) Schematic of strategy for replacing both LC1 alleles with an LC1 mutant transgene. Asterisks represent the K203A and R210A substitutions in LC1. Gray arrows show position of primers used for PCR in panel b. (**b**) PCR amplification of the LC1 locus from parental cells (WT), single knock-in cells (SKI) and double knock-in cells (DKI). Knock-in constructs are larger than WT owing to presence of the drug resistance marker and intergenic region. Primer positions are shown in panel a. Right side shows sequence of codons 203 and 210 of the LC1 gene in parental cells (WT) and the double knock-in (DKI). (**c**) Mean squared displacement (MSD) was determined for the indicated cell lines as described in Methods. Bold lines represent the mean MSD of two biological replicates from two independent experiments (>70 cells tracked per line per experiment for a total of >175 cells tracked per cell line). Dashed lines show the standard error of the mean between two independent replicates. Inset shows growth curve of the indicated cell lines in culture. Error bars show standard deviation of three replicates. For WT and DKI, standard deviation is smaller than the size of the symbols.

To generate a constitutive motility mutant completely lacking WT LC1, we used homologous recombination to replace both LC1 alleles with the HA-tagged LC1-K203A/R210A mutant transgene that was used in the RNAi-K/R mutant^16^ (Fig. 1a). PCR amplification of the LC1 locus and sequencing of the open reading frame demonstrated the resulting double knock-in, “DKI”, is homozygous for the K203A/R210A mutation (Fig. 1b, Supplementary Fig. S7). Growth of the DKI mutant is somewhat reduced in culture, though not as markedly as in the RNAi-K/R mutant (Fig. 1c, inset). Motility analysis shows the DKI mutant is essentially devoid of propulsive motility and the defect is more pronounced than that observed for the RNAi-K/R mutant (Fig. 1c, Supplementary Fig. 1).

### Parasite motility is critical for virulence

**Figure 2:**
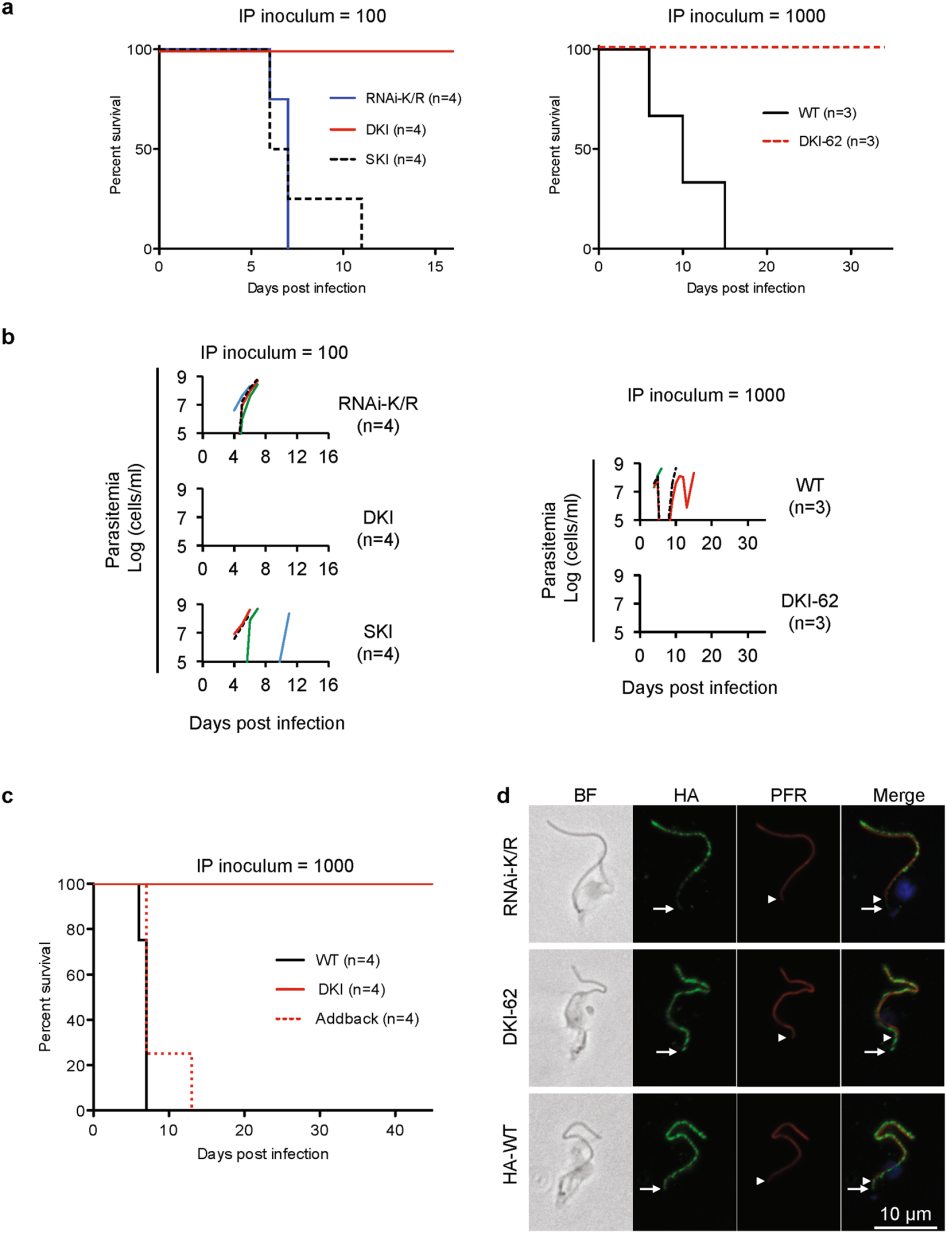
Virulence defect of LC1 DKI motility mutants. (**a**) Survival curves for mice infected intraperitoneally with the indicated cell lines. Graphs show data from two independent experiments, RNAi-K/R vs DKI vs SKI (inoculum = 100 parasites, n = 4 mice each) and WT vs DKI-62 (inoculum = 1000 parasites, n = 3 mice each). Data are representative of the phenotypes observed for WT, RNAi-K/R and DKI cell lines across multiple independent experiments (Figs 2c, 3c, and data not shown)^17^. DKI and DKI-62 are independently derived LC1 K203A/R210A double knock-in mutants. (**b**) Parasitemia of mice used for panel a. Parasitemia in blood was measured beginning four days post infection. Detection limit is ∼1e5 cells/ml. (**c**) Survival curves for mice infected intraperitoneally with the indicated cell lines. Addback is a DKI mutant line into which a WT copy of LC1 has been introduced at an ectopic locus, as described in Methods. (**d**) Immunofluorescence microscopy of detergent-extracted cytoskeletons. HA-tagged LC1 (green), PFR (red), DAPI (blue). The white arrows and arrowheads mark the proximal ends of LC1 and PFR staining, respectively. RNAi-K/R cells were examined 72 h after Tet-induction^16,17^. Images were collected under identical conditions and processed identically.

We next compared mouse infection by DKI and RNAi-K/R mutants (Fig. 2a,b). Consistent with our previous studies^17^, mice infected intraperitoneally with WT *T. brucei* or the RNAi-K/R mutant show rapid onset of parasitemia and lethal outcome in less than 14 days (Fig. 2a,b). By contrast, mice infected with either of two independent DKI mutants survived through the experimental endpoint and never showed detectable parasitemia (Fig. 2a,b). Surviving mice were protected against subsequent infection with WT *T. brucei* expressing the surface glycoprotein VSG221 (not shown), verifying mice were indeed exposed to the DKI mutant, which also expresses VSG221.

We hypothesize that the difference in virulence between RNAi-K/R and DKI mutants is due to mixed expression of WT and mutant LC1 in the RNAi-K/R mutant resulting from incomplete knockdown^16^. In support of this hypothesis, virulence was fully restored in an “Addback” line in which WT LC1 was introduced into the DKI mutant (Fig. 2c). As an independent test, we examined single knock-in parasites, “SKI”, which have one WT and one mutant copy of LC1. Mice infected with SKI parasites showed parasitemia and survival similar to that obtained with infection by WT and RNAi-K/R parasites (Fig. 2a,b). Therefore, mixed expression of WT and mutant LC1 suffices for WT virulence.

To test whether the virulence defect of the DKI mutant was due to poor expression or assembly of the LC1 mutant protein on the axoneme, we performed immunofluorescence microscopy on detergent-extracted cytoskeletons. As anticipated, the mutant LC1 protein is stably associated with the flagellum in both RNAi-K/R and DKI mutant cells (Fig. 2d). In both cases, the LC1 signal extends beyond the proximal end of the paraflagellar rod (PFR), as observed for WT LC1 protein, indicating it is on the axoneme. Viability of both mutants (Fig. 1c, inset) is also consistent with proper assembly into outer arm dyneins on the axoneme, because loss of LC1 disrupts outer arm dynein and is lethal in bloodstream form *T. brucei*^10^. Therefore, our combined results demonstrate that blocking propulsive motility through disrupting LC1 function severely impairs parasite virulence.

### Modification of the LC1 N-terminus blocks propulsive motility

**Figure 3:**
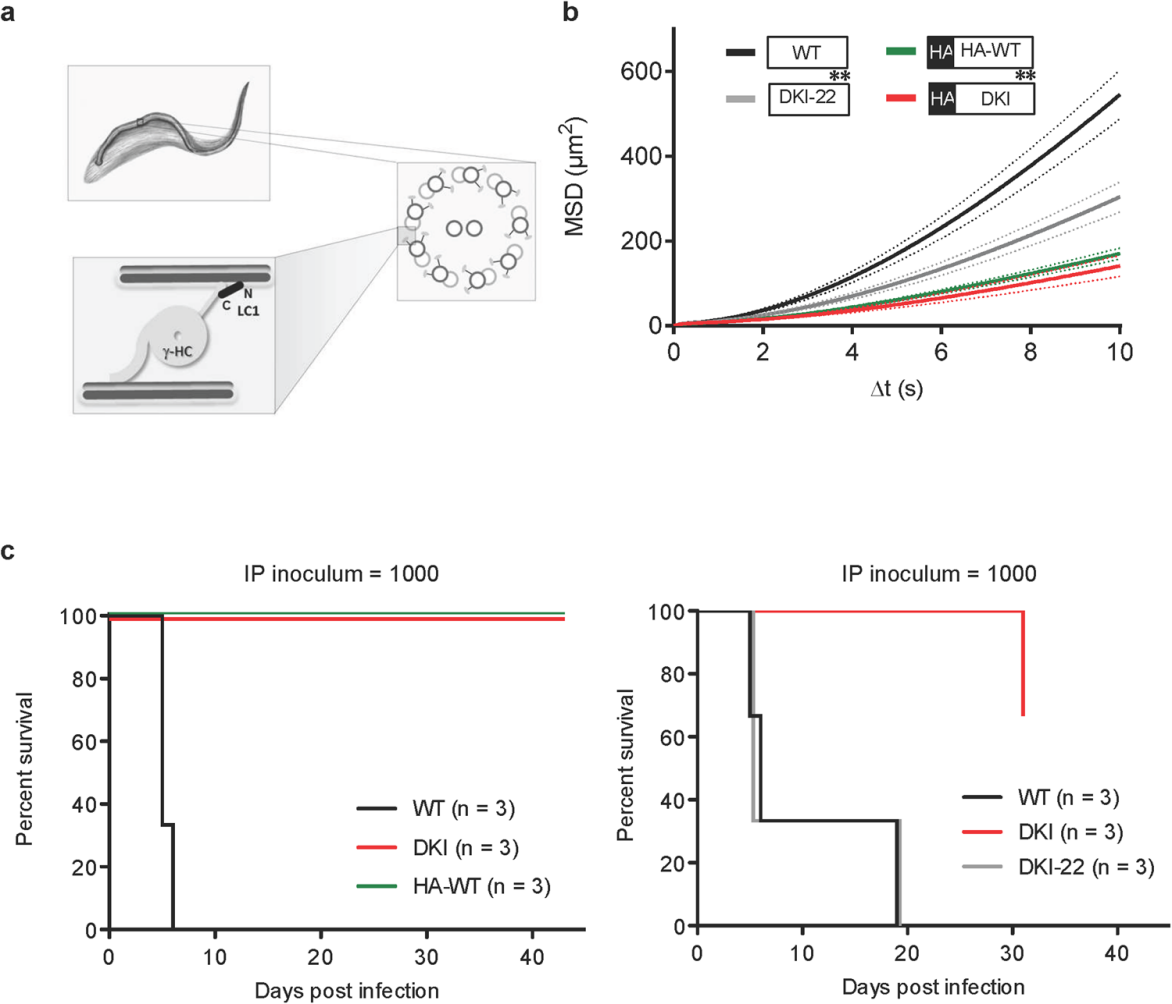
The LC1 N-terminus is important for motility. (**a**) Drawing depicts the flagellar axoneme of *T. brucei* and model for LC1 function with C-terminus (C) and N-terminus (N) contributing to interaction with the outer dynein gamma heavy chain (γ-HC) and microtubule doublet, respectively. Based on^19,20^. (**b**) Mean squared displacement (MSD) was determined for the indicated cell lines as described in Methods. Bold lines represent the average MSD across seven biological replicates from three independent experiments (>400 cells tracked per cell line per replicate for a total of >4200 cells tracked per cell line). Dashed lines represent the standard error of the mean between seven replicates. Inset shows the HA-tag and point mutant status of LC1 in each cell line. Asterisks represent K203A and R210A substitutions in LC1. (**c**) Survival curves for mice infected with the indicated cell lines. The HA-tag and point mutant status are as indicated in the panel b inset.

Having established a requirement for LC1 and motility in infection, we next used the trypanosome system to examine mechanism of LC1 function. *In vitro* studies suggest a model in which LC1 controls sliding of adjacent axonemal microtubule doublets through electrostatic interactions between microtubules and basic residues in the LC1 N-terminus (Fig. 3a)^19,20^. However, importance of the LC1 N-terminus has not been tested in live cells. The DKI LC1 transgene contains an N-terminal HA-tag with several acidic residues that are expected to affect electrostatic interactions with microtubules. To test the importance of the LC1 N-terminus, we therefore examined motility and virulence of parasites expressing LC1 with or without an N-terminal HA tag. Motility was more severely impaired in the HA-tagged mutant (DKI) than it was in the untagged mutant (DKI-22) (Fig. 3b). In fact, we found that the HA tag alone was sufficient to block LC1 function, as motility of HA-WT cells was indistinguishable from motility of tagged DKI mutants (Fig. 3b, Supplementary Fig. S1). Consistent with our earlier observations (Figs 1 and 2), parasites with severe motility defects (HA-WT or DKI) were avirulent while those retaining some motility (DKI-22) infected mice like WT (Fig. 3c, Supplementary Fig. S2). These results support the *in vitro* model^19,20^ for a requirement of the LC1 N-terminus in axonemal motility and provide additional support for the requirement of trypanosome motility for virulence.

### Motility is critical for evasion of the host immune response during bloodstream infection

**Figure 4:**
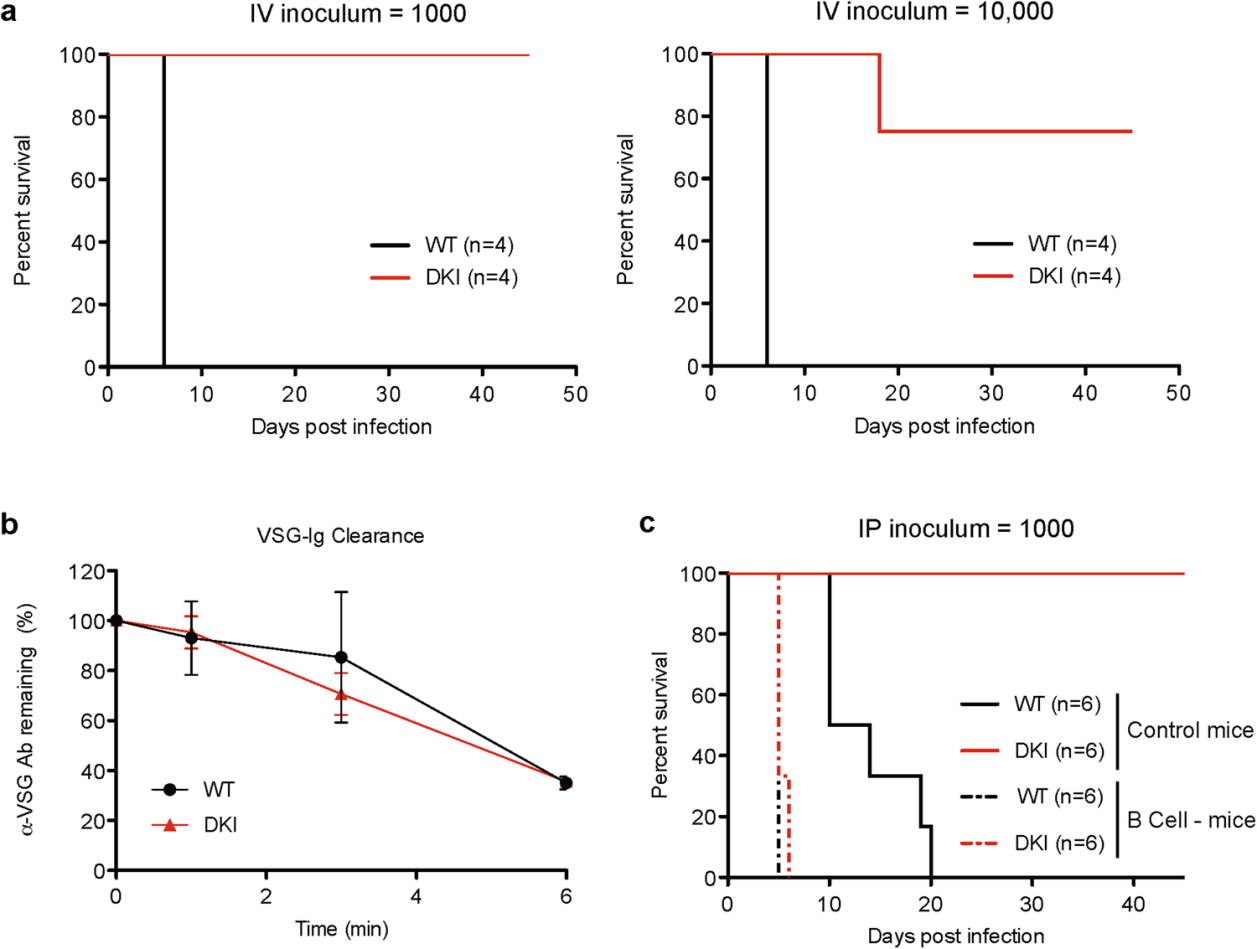
The LC1 DKI motility mutant is defective in establishing and maintaining bloodstream infection. (**a**) Survival curves for mice infected intravenously with the indicated cell lines. Parasitemias are shown in Supplementary Fig. S3. (**b**) Time course of VSG-Ig clearance after shift to 37 °C. Error bars show standard error of the mean of three replicates. (**c**) Survival curves for control mice and mice lacking B cells (B cell−) that were infected intraperitoneally with the indicated trypanosome cell lines (WT or DKI). Data are from two independent experiments (n = 3 mice per experiment). Parasitemias from one of the experiments are shown in Supplementary Fig. S4.

We next explored potential explanations for the virulence defect of motility mutants. One possibility is that loss of motility impedes parasite entry into the bloodstream after intraperitoneal infection. To address this question, parasites were introduced directly into the bloodstream by intravenous infection. A pronounced virulence defect was seen, even when parasites were delivered intravenously at an inoculum of 1,000 or 10,000 parasites per mouse (Fig. 4a). Parasites were confirmed to be present in the bloodstream for five of eight intravenous infections in two independent experiments, and detectable parasitemia was cleared by 16 days post infection in all but one infection (Supplementary Fig. S3). Survival was enhanced for all mice infected with the DKI mutant and seven of eight mice survived through the experimental endpoint, remaining free of detectable parasites. Therefore, parasite motility is critical for establishment and maintenance of infection within the bloodstream, rather than simply for accessing the bloodstream from an extravascular site.

It has been proposed that trypanosome forward motility might contribute to survival in the bloodstream by driving movement of host antibodies bound to variant surface glycoprotein (VSG-antibody complexes) to the cell posterior for endocytic clearance^21^. We therefore examined clearance of VSG-antibody complexes in the DKI mutant, but did not observe any differences compared to WT cells (Fig. 4b), indicating the virulence defect is not due to an inability to clear VSG-antibody complexes. This result prompted us to consider the possibility that the virulence defect might result from failure to meet nutritional requirements in the bloodstream, rather than failure to avoid the host immune response. For example, endocytosis in trypanosomes is restricted to the flagellar pocket^22^ and defective flagellar motility might therefore alter access to macromolecular nutrients^23^. To test this, we asked whether DKI mutants were able to infect mice with a defective immune system. As shown in Fig. 4c, DKI mutants mounted a lethal infection in B cell mutant mice that was indistinguishable from infection by WT parasites in terms of parasitemia and survival (Fig. 4c, Supplementary Fig. S4). Therefore, an intact host immune system is required to control infection.

### Influence of host environment on parasite motility

To date, most studies have employed cultured cells to assess trypanosome motility although efforts have been made to simulate blood conditions, e.g. increasing viscosity, adding blood to cultured cells, or engineering artificial obstacles to movement^17,21,24,25^. Nonetheless, such experiments cannot replicate the complex host environments encountered during infection, where high viscosity, non-Newtonian fluid properties and host responses to infection will influence parasite movement. We therefore examined movement of WT and LC1 mutant parasites directly in whole blood from infected mice.

High density of red blood cells makes it difficult to analyze trypanosome motility in undiluted blood. To overcome this limitation, we infected mice with mCherry-expressing parasites and examined parasite motility *ex vivo*, in whole blood taken directly from infected mice. Importantly, expression of mCherry did not alter the course of infection (Supplementary Fig. S5). Individual parasites were well-resolved using real-time fluorescence video microscopy, even amidst densely-packed red blood cells (Supplementary Video S1, Supplementary Fig. S6). Despite having a beating flagellum, DKI mutants showed no translocation (Supplementary Videos S2, S3), corroborating the absence of propulsive motility observed *in vitro*. For WT parasites, cell movement was highly variable, making it unrealistic to assign a general description that applies to all cells (Supplementary Videos S4, S5). Notably, WT parasites usually did not show sustained unidirectional translocation as predicted by previous studies in culture medium^17,21,24,25^. Instead, most cells remained very close to the point of origin, showing oscillating movements back and forth. Some cells showed short intervals of translocation, either with the flagellum tip leading (forward) or trailing (backward), but this was usually saltatory and interspersed with pauses and reversals (Supplementary Videos S4, S5).

## Discussion

Motility of trypanosome cells is distinctive in many ways from that of other flagellated eukaryotic cells^2,3,25–27^ and the genus name is in fact derived from the organism’s hallmark auger-like motility^28^. This form of motility has been postulated to present advantages for movement and survival of the parasite within the mammalian host during infection^21,25,29,30^. Nonetheless, despite being a focus of intense study for many years, unequivocal evidence of a requirement for trypanosome motility during infection of a mammalian host has been lacking. Our studies therefore provide an important advance by demonstrating a critical requirement for *T. brucei* motility during mouse infection. To our knowledge, this is the first virulence defect in any protozoan pathogen that can be specifically attributed to loss of dynein-dependent flagellar motility. Importantly, we demonstrate this defect is due to disruption of protein function rather than removal of flagellar proteins, which is invariably lethal^15^. These results are particularly significant from a therapeutic standpoint, because protein levels are challenging to manipulate with small molecules, while inhibiting protein activity is more readily achieved. Given the recent development of potent and specific dynein inhibitors^31–33^, together with a distinctive axonemal dynein repertoire in *T. brucei*^34,35^, our findings open the possibility of targeting dynein activity for therapeutic intervention in trypanosome infections.

Recent work has shown that *T. brucei* in extravascular compartments such as dermal and adipose tissue may contribute significantly to pathogenesis and transmission^36–38^. Our studies demonstrate a requirement for motility in bloodstream infection, but do not rule out the possibility that motility mutants might reside in extravascular compartments. It will be important for future studies to address this question, particularly in the context of therapeutic efforts to eliminate infection.

We were initially surprised by the dramatic difference in virulence between the RNAi-K/R mutant and the DKI mutant. However, while the RNAi mutant shows a clear motility defect relative to WT cells, qRT-PCR demonstrates that mRNA expression is reduced by only ∼60%^16^. This leaves a substantial amount of residual WT LC1 that could be incorporated into the axoneme and support residual propulsive motility and infectivity. There is somewhat weaker axonemal staining for LC1 in the RNAi-K/R mutant compared to the DKI mutant (Fig. 2d), consistent with residual WT LC1 competing for incorporation into the axoneme. Furthermore, the SKI and Addback mutants each express WT and mutant LC1 and virulence of these lines is indistinguishable from WT, lending independent support to the idea that mixed expression of WT and mutant LC1 suffices to support WT virulence. Complete removal of WT protein in the DKI mutant, on the other hand, leads to a correspondingly greater block in propulsive motility and a concomitant defect in infectivity.

RNAi-K/R and DKI-22 mutants exhibit motility intermediate between that of DKI and WT (Figs 1c,3b, Supplementary Fig. 1), yet they show parasitemia and infection outcome indistinguishable from WT (Figs 2a,b, 3c, Supplementary Fig. S2). This result suggests that successful bloodstream infection exhibits a threshold dependence on propulsive motility, such that completely normal motility is not required, but if motility drops below a minimal threshold, the host is able to successfully defeat the infection. Consistent with this idea, B cell mutant mice were unable to clear infection by motility mutants, demonstrating the host immune system is required for preventing infection. Our results do not rule out the possibility that some feature of motility other than cell propulsion contributes to the virulence difference between mutants, but there is a clear correlation with cell propulsion.

Numerous studies using cells in culture have indicated that forward translocation is a prominent mode of trypanosome motility and that increasing viscosity or obstacle density to mimic blood enhances sustained forward translocation^17,21,25^. Movement of parasites within whole blood is less characterized. In a pioneering study, Bargul and colleagues examined *T. brucei* motility in blood smears prepared from mice that were immunosuppressed^24^. They observed sustained translocation (≥16 s) for ∼30% of cells, while another ∼45% of cells exhibited translocation interrupted by at least one ‘tumbling’ phase during which there was little net movement. However, they also observed reversals between forward and backward movement for many cells. We examined both WT and DKI mutants in whole blood taken directly from infected mice (Supplementary Videos S2–S5). As expected, DKI mutants were unable to translocate. Consistent with the Bargul study we observed frequent reversals between forward and backward movement for WT cells, although we saw fewer clear examples of sustained translocation. Rather, most WT cells in our analysis remained near their point of origin, despite short oscillating movements. Differences might reflect use of blood films in the earlier study^24^, which spread samples out to allow visualization of individual cells but make translocation mostly two-dimensional, or differences in trypanosome strains used. Additionally, our samples were taken from immunocompetent animals, so parasites are under attack by the host immune system and this might impact cell movement. Nonetheless, in both studies, rapid back and forth movements are more prominent than suggested from earlier *in vitro* studies. Recent work has shown that trypanosome flagellar waveform and cell motility are modified by changes in the extracellular environment^24,25^. Therefore, differences observed in blood versus cell culture medium likely reflect the unique microenvironment of blood. The bloodstream of an infected host provides additional features that are not captured even in whole blood samples, e.g. confinement within vessels and fluid flow. Nonetheless, our results emphasize the complexity of the blood environment and the need for further analysis of parasite movements directly in blood of an infected host.

Although the precise role for parasite motility during infection is not yet clear, motility is required to avoid some aspect of the host immune response, because motility mutants efficiently infect B cell mutant mice, but not WT mice. (Fig. 4c, Supplementary Fig. S4). This result potentially implicates parasite motility in avoidance of antibody-mediated immune responses, although a complete lack of B cells could impact other aspects of host immune function. Recent studies revealed that rapid clearance of *T. brucei* cells following VSG knockdown during infection *in vivo*^39^ is correlated with enhanced phagocytosis of antibody-bound parasites by macrophages *in vitro*^40^. Interestingly, increased phagocytosis of these VSG RNAi cells is also associated with reduced tendency of parasites to change direction of movement^40^. We observed that sustained cell translocation was not a prominent feature in whole blood from an infected host but rapid back and forth movements were common. Therefore, one role for motility might be to provide short bursts of increased cell speed or changes in direction to actively avoid host immune cells. Interestingly, trypanosomes modulate the host immune response by presenting VSG, either as a membrane-associated form (mfVSG) attached to the parasite surface, or as a soluble form (sVSG) that is shed during infection^41^. mfVSG is reported to facilitate more robust macrophage activation^41^ and reduced production of sVSG leads to attenuated virulence^42^. If motility were functioning to avoid host immune cells, loss of motility in the DKI parasites would increase the relative presentation of mfVSG versus sVSG and this might contribute to the ability of mice to control infection by this mutant.

LC1 is found in essentially all eukaryotic organisms with a motile flagellum^43,44^ and has been implicated in human diseases caused by flagellum defects^45^. The LC1 C-terminus binds directly to dynein on one axonemal microtubule doublet while the LC1 N-terminus interacts with the adjacent microtubule doublet (Fig. 3a)^19,20^. These interactions are proposed to modify dynein activity and sliding resistance between adjacent microtubule doublets, but a role for the LC1 N-terminus has not been demonstrated in living cells. By taking advantage of facile molecular genetics in *T. brucei*, we examined multiple LC1 variants and our results provide *in vivo* support for the proposed model of LC1 function in flagellar motility (Fig. 3a)^19,20^. Therefore, in addition to the importance for *T. brucei* pathogenesis, our findings are relevant for understanding mechanisms of dynein regulation across diverse organisms, including its role in heritable human diseases.

Our combined studies identify parasite motility as a virulence factor for trypanosome infection and provide insight into mechanisms of LC1 function. Importantly, although fundamental mechanisms of flagellar motility are conserved across diverse species, protozoan parasites exhibit numerous lineage-specific features and machinery^2,3,44,46^, thereby presenting avenues for specifically targeting motility of these pathogens.

## Methods

### Trypanosome cell lines and cell culture

All trypanosomes were derived from VSG 221-expressing, bloodstream single marker (BSSM) cells and cultivated as described^47^. The RNAi-K/R mutant was described previously as LC1-K203A/R210A (K/R)^16,17^ and was grown in the presence of 1 µg/ml tetracycline to induce RNAi knockdown of endogenous LC1 and expression of the K/R point mutant as described^16,17^. For generating the DKI, SKI, DKI-22 and HA-WT cell lines, parasites were stably transfected with the appropriate integration cassettes in the pMOTag^48^ vector backbone. Integration cassettes contain the HA-tagged LC1 coding sequence (WT or LC1-K/R^16^), together with a drug marker, and flanked by sequences corresponding to the LC1 5′ (997 bp) or 3′ (887 bp) untranslated regions to direct integration as depicted in Fig. 1a. The blasticidin resistance gene is from pTUB-Blast^49^. All constructs were verified by sequencing. Stable transfection was done as described^47^. Presence of the K/R mutation and HA tag in the corresponding cell lines was verified by PCR-amplification of the LC1 locus and sequencing. The DKI-22 mutant was identified as a cell line in which integration occurred within the LC1 open reading frame, thus omitting the N-terminal HA tag. mCherry expressing parasites were generated by stable transfection with the pNKmCherry plasmid^17^. To generate the LC1 addback line, the mCherry gene in pNKmCherry^17^ was replaced with the wild type LC1 gene and the resulting plasmid was linearized and transfected into the DKI mutant. For growth curves, cell densities were measured using a Z1 Coulter counter (Beckman) for three independent cultures grown in parallel.

### Primers for PCR and sequencing

Primers for amplification of the LC1 locus (gray arrows in Fig. 1a) were: LC15′Forward: GAGTGGGGGTAAATCAGCAT; LC13′Reverse: TTCCATCGAGGTTTGGTTTT. For amplifying each LC1 allele independently, the LC15′Forward primer was used with reverse primers specific to either the blasticidin: BlastRev: TAGCCGTTGCTCTTTCAATGA, or the phleomycin: PhleoRev: GAACGGCACTGGTCAACT resistance genes. Sequencing of the 5′ and 3′ ends of the LC1 gene was done using primer: LC1orfrev: ATTTGTGGAGAGCGCAAGGT and primer: LC1orffor: TCGCACGATGGAGATGACAG, respectively.

### Immunofluorescence microscopy of detergent-extracted cytoskeletons

Cytoskeletons were prepared for immunofluorescence by detergent extraction with 1% NP-40 as previously described^50^. Briefly, cytoskeletons were prepared in solution then settled onto poly-L-lysine-coated coverslips for 15 min. After removal of unattached material, samples were fixed with 2% paraformaldehyde in PBS for 15 min. Coverslips were washed and blocked in PBS + 8% normal donkey serum +2% BSA, followed by incubation with the following primary antibodies diluted in blocking solution: mouse anti-HA.11 (1:200, Biolegend) and rabbit anti-PFR2 (1:1000^51^). Anti-mouse and anti-rabbit secondary antibodies conjugated to Alexa 488 and Alexa 594, respectively, were added at 1:1500 dilution. Coverslips were mounted in Vectashield with DAPI and images were acquired on a Zeiss Axioskop II compound microscope with a 100x, 1.4 NA objective-lens and Axiovision software. Images shown in Fig. 2c were all acquired with the same exposure settings and processed identically using Adobe Photoshop.

### Parasite motility analyses

Motility assays were performed in pre-warmed motility chambers as described^16,52^ with the following modifications. Chambers were 70–100 µm thick. For Fig. 1c and Supplementary Fig. 1a, mCherry-expressing trypanosomes were centrifuged and resuspended in fresh culture medium then equilibrated for 5 min at 37 °C with 5% CO_2_ before loading chambers. 10 s videos were recorded by fluorescence microscopy with a 20x, 0.4 NA objective-lens. For Fig. 3b and Supplementary Fig. 1b, 30 s videos of cultured trypanosomes were recorded under dark-field illumination with a 10x, 0.30 NA objective-lens. Videos were recorded at 30 frames/s on a Zeiss Axiovert 200 M inverted microscope using Adobe Premiere Elements 9 and each chamber was imaged for a maximum of ∼5 min.

An automated multiple-particle tracking algorithm was developed in MATLAB based on the u-track algorithm^53^ and optimized for the specific detection and tracking of trypanosomes. Trypanosomes in fluorescence video frames were segmented by using a threshold, and then the 2-dimensional coordinates of their centers-of-mass across different frames were linked to each other to generate the corresponding trajectory. For dark-field microscopy based video analysis, adjacent-frame subtraction and averaging was applied such that the moving parasites (either moving directionally or “wiggling” locally) could be located. @msdanalyzer MATLAB class was then used to calculate the mean squared displacement (MSD) for each trajectory^54^. The mean MSD of trajectories from independent biological replicates of each cell line are plotted in Figs 1c,3b, and Supplementary Fig. 1, as described in the corresponding figure legends.

### Mouse infections

All methods were carried out in accordance with the guidelines and regulations of the UCLA Institutional Animal Care and Use Committee (IACUC), NIH Public Health Service Policy on Humane Care and Use of Animals, USDA Animal Welfare regulations, and AAALAC International accreditation standards under IACUC-approved protocol ARC# 2001-065. Mouse infections to assess parasite virulence were performed as described^17^ with the following modifications: 6–10 weeks old female mice (Jackson Laboratory) were injected intraperitoneally (IP) with 100 or 1,000 parasites in 0.2 ml ice-cold phosphate buffered saline +1% glucose (PBS-G) or intravenously (IV) via the tail vein with 1,000 or 10,000 parasites in 0.1 ml PBS-G. Parasitemia was monitored by counting in a hemocytometer beginning 3–4 days post infection, and mice were euthanized if parasitemia exceeded 2e8 cells/ml. All mice were BALB/c (Jackson Laboratory, JAX 000651) except for Fig. 4c and Supplementary Fig. S4, which used C57BL/6 for WT (Jackson Laboratory, JAX 000664) and Bcell- (*Ighm*^*tm1Cgn*^) in C57BL/6 background (Jackson Laboratory, JAX 00288).

### Video microscopy of parasites in whole blood from infected mice

Female BALB/c mice (Jackson Laboratory, JAX 000651) were injected intraperitoneally with 100,000 or 200,000 mCherry-expressing parasites. Mice were euthanized when parasitemia reached ∼0.5–1e7 cells/ml (2–3 days post infection). Blood was collected by cardiac puncture and transferred into heparinized collection vials to prevent clotting. Heparinized blood samples were incubated at 37 °C with 5% CO_2_ for 5 min before loading into pre-warmed poly-glutamate coated chambers. Videos were recorded within 15 min of blood collection by fluorescence microscopy as described above.

### VSG-Ig clearance

VSG-Ig clearance assay was performed as described^55^.

### Data availability

All data generated or analysed during this study are included in this published article (and its Supplementary Information files).

## Electronic supplementary material

Supplementary Video S1

Supplementary Video S2

Supplementary Video S3

Supplementary Video S4

Supplementary Video S5

Supplementary Information
