# Supplementary material for: Parasite motility is critical for virulence of African trypanosomes
Source: Sci Rep. 2018 Jun 14;8:9122. doi: 10.1038/s41598-018-27228-0 (PMC6002391; doi:10.1038/s41598-018-27228-0)
Supplement: Supplementary file 6 — Supplementary Information [file 41598_2018_27228_MOESM6_ESM.pdf]

Supplementary material for

Parasite motility is critical for virulence of African trypanosomes

Michelle M. Shimogawa[1], Sunayan Ray[1,6], Neville Kisalu[1,7], Yibo Zhang[2,3,4], Quanjie Geng[2], Aydogan Ozcan[2,3,4] and Kent L. Hill[1,4,5,*]

Supplementary Figure S1

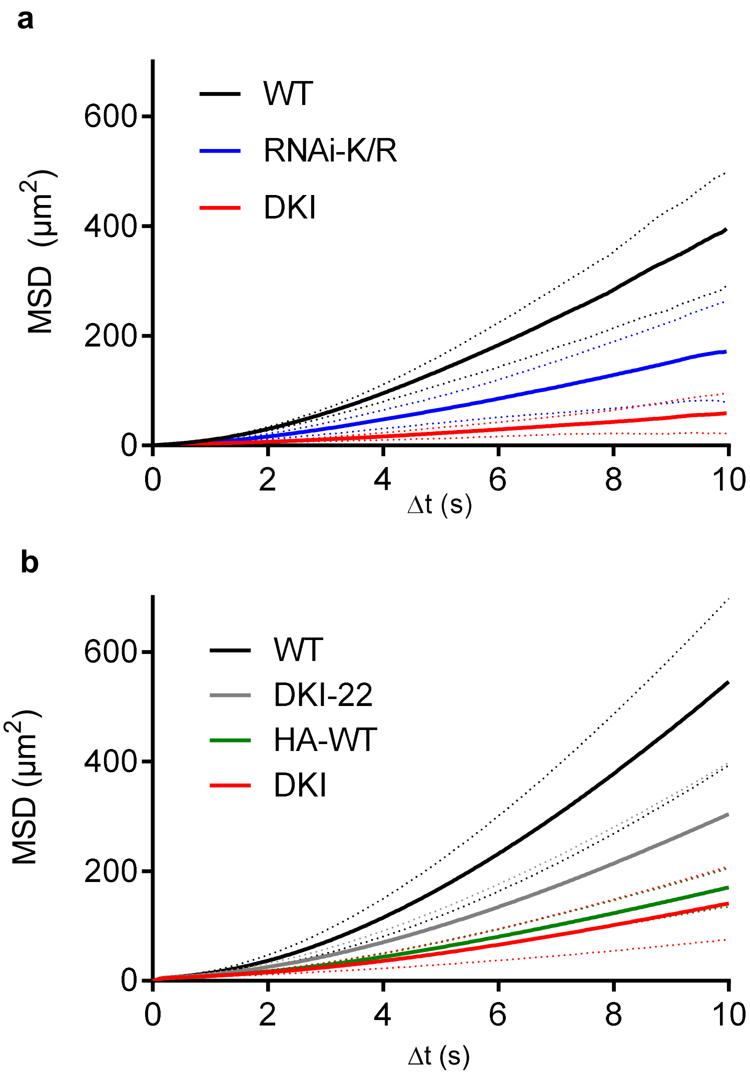

Supplementary Figure S1. Motility analysis of LC1 mutants. Mean squared displacement (MSD) was determined for the indicated cell lines as described in Methods. (a) Graph shows the same data as Fig. 1c except dashed lines show the standard deviation between replicates. Data are from 2 biological replicates from 2 independent experiments (n=2). (b) Graph shows the same data as Fig. 3b except dashed lines show the standard deviation between replicates. Data are from 7 biological replicates from 3 independent experiments (n=7).

Supplementary Figure S2

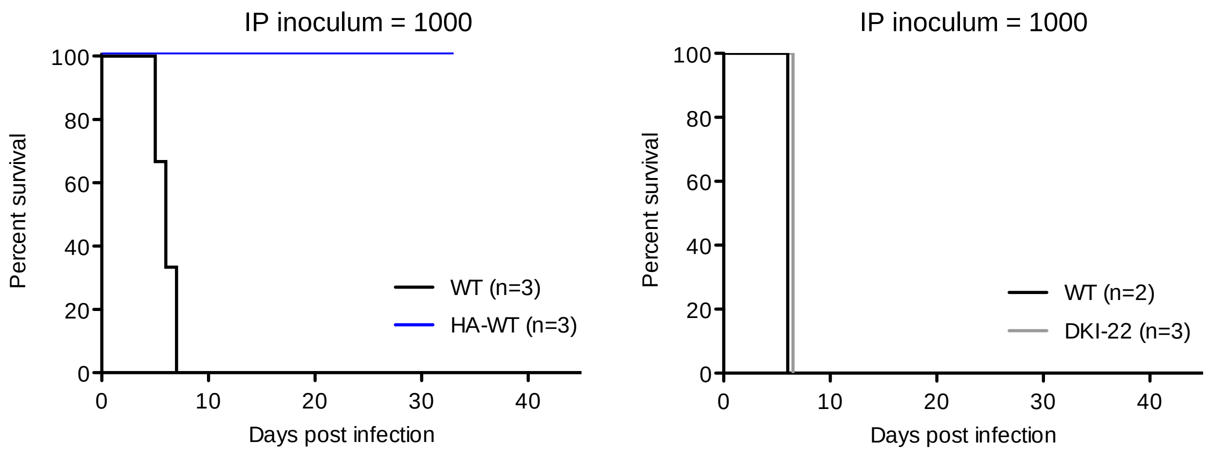

Supplementary Figure S2. Mouse infections with HA-WT and DKI-22 parasites. Survival curves for mice infected intraperitoneally with the indicated trypanosome cell lines.

Supplementary Figure S3

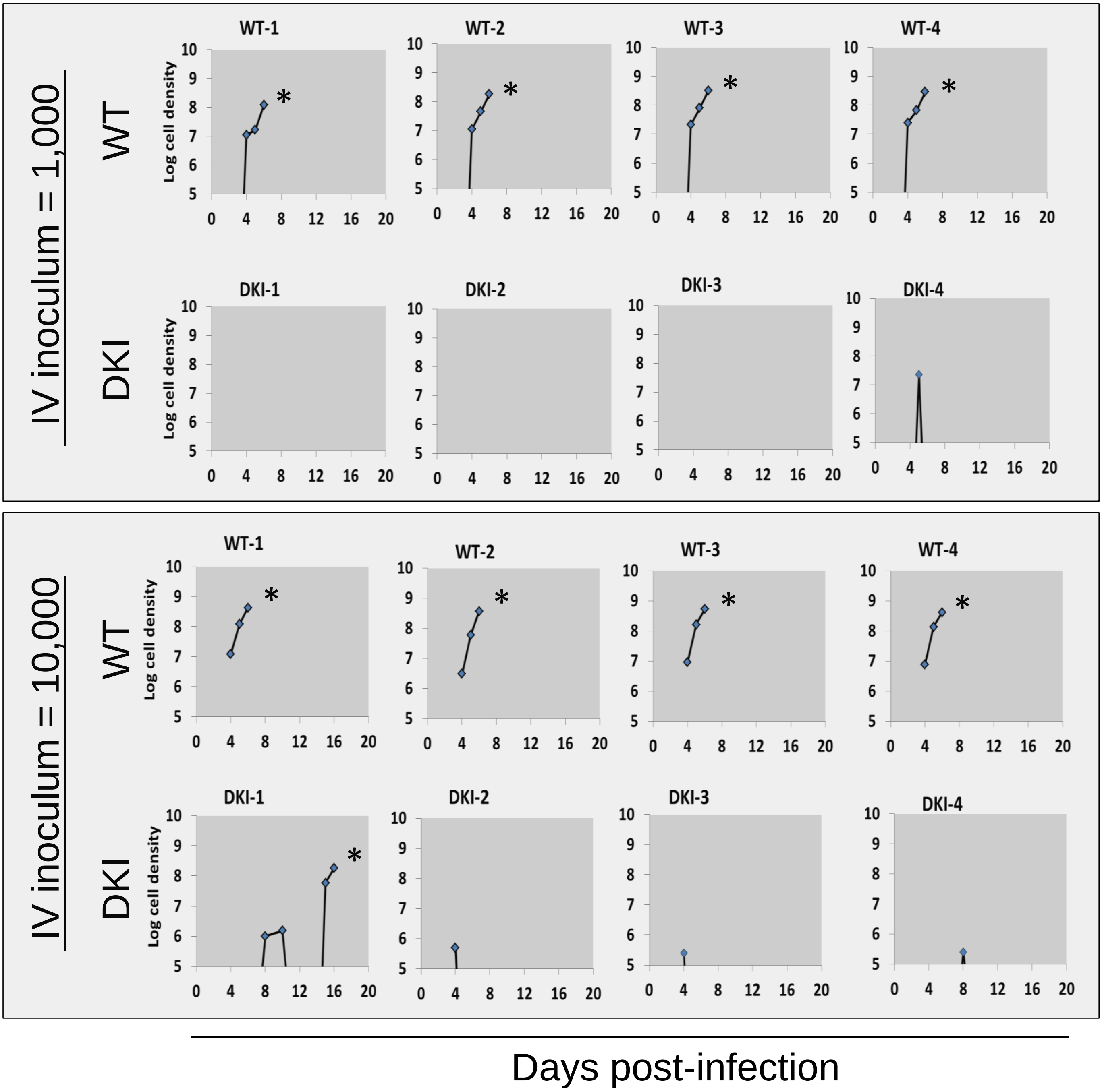

Supplementary Figure S3. Parasitemia of mice infected intravenously with WT or DKI parasites. Parasite inoculum is 1,000 or 10,000 as indicated. Parasitemia in blood was measured beginning 3-4 days post infection. Detection limit is ~1e5 cells/ml. Asterisks indicate mice were sacrificed when parasitemia exceeded 2e8 cells/ml.

Supplementary Figure S4

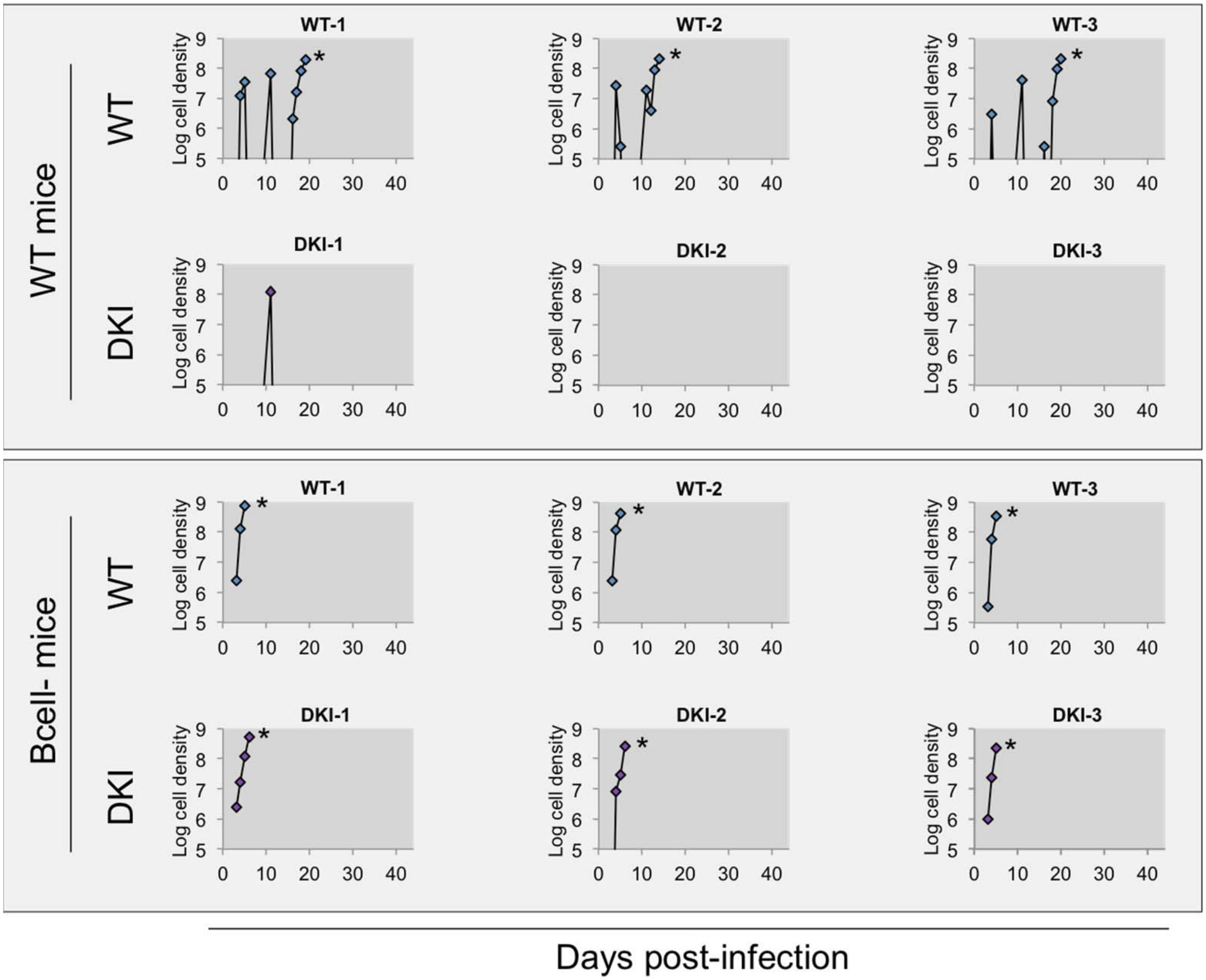

Supplementary Figure S4. Parasitemia of Bcell- mice infected intraperitoneally with WT or DKI parasites. WT (C57BL/6) or Bcell- mice (*Ighm*^{tm1Cgn}) were infected intraperitoneally with 1000 parasites as indicated. Parasitemia in blood was measured beginning 3-4 days post infection. Detection limit is ~1e5 cells/ml. Asterisks indicate mice that succumbed or were sacrificed when parasitemia exceeded 2e8 cells/ml.

Supplementary Figure S5

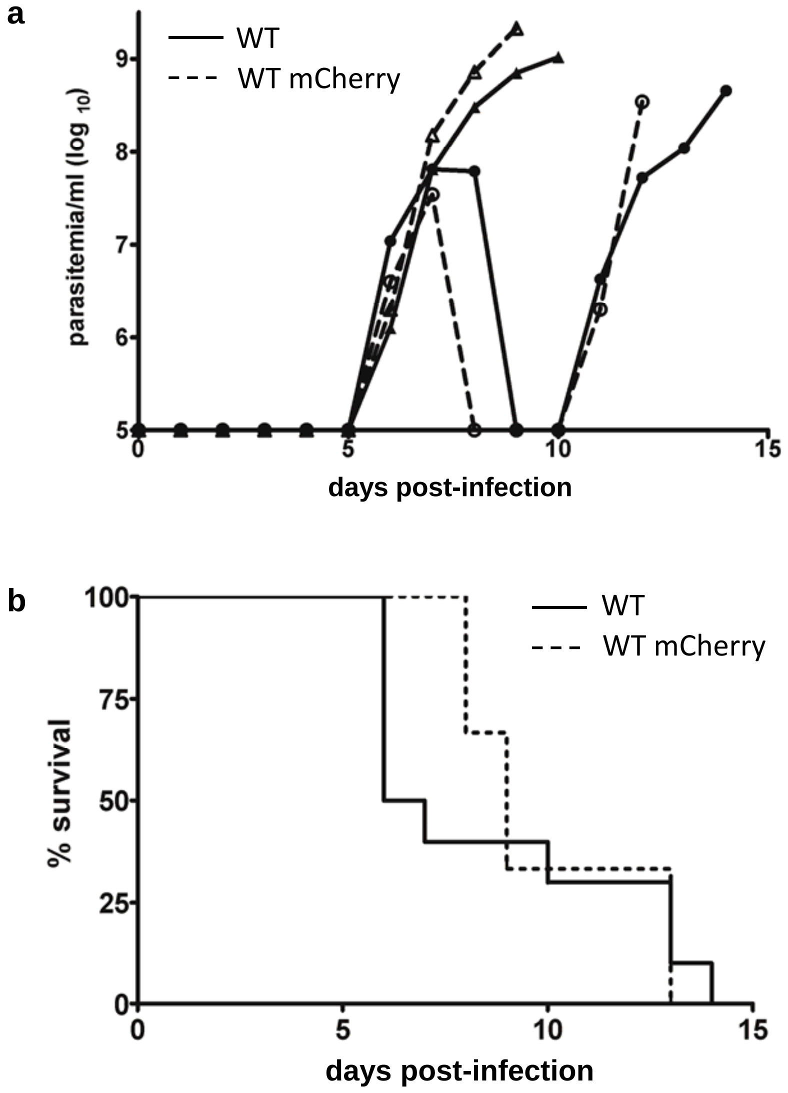

Supplementary Figure S5. Mouse infections with parasites expressing mCherry. (a) Parasitemia of mice infected intraperitoneally with 100 WT (solid lines) or mCherry-expressing (dashed lines) parasites. Detection limit is ~1e5 cells/ml. (b) Survival curves for mice infected as described in panel a.

Supplementary Figure S6

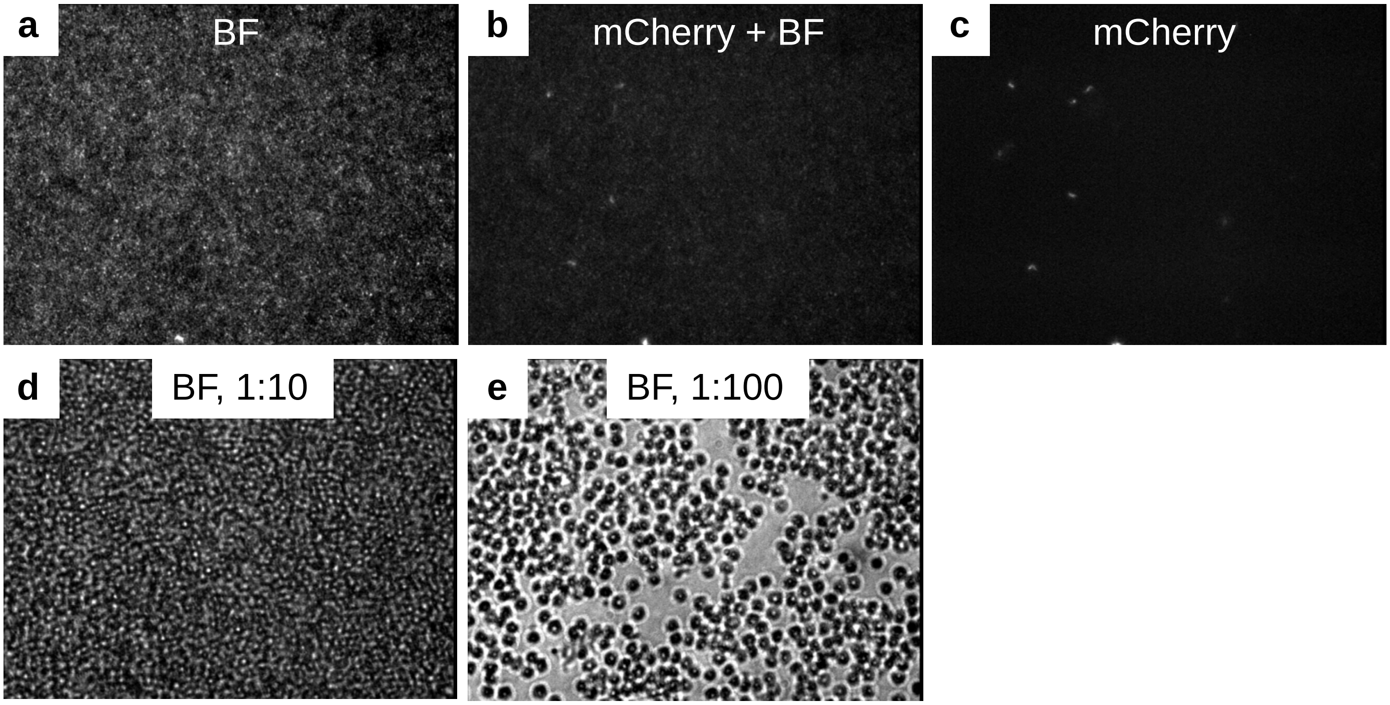

Supplementary Figure S6. Mouse infections with parasites expressing mCherry. (a-c) Still frames from live video microscopy (Supplementary Video S1) of undiluted blood from a mouse infected with mCherry *T. brucei* showing bright field (BF) illumination of red blood cells, fluorescence (mCherry) illumination of parasites, or simultaneous illumination of both (mCherry + BF). Parasites are not visible without fluorescence. (d-e) 1:10 and 1:100 dilutions of mouse blood to illustrate the density of red blood cells in undiluted samples.

Supplementary Figure S7

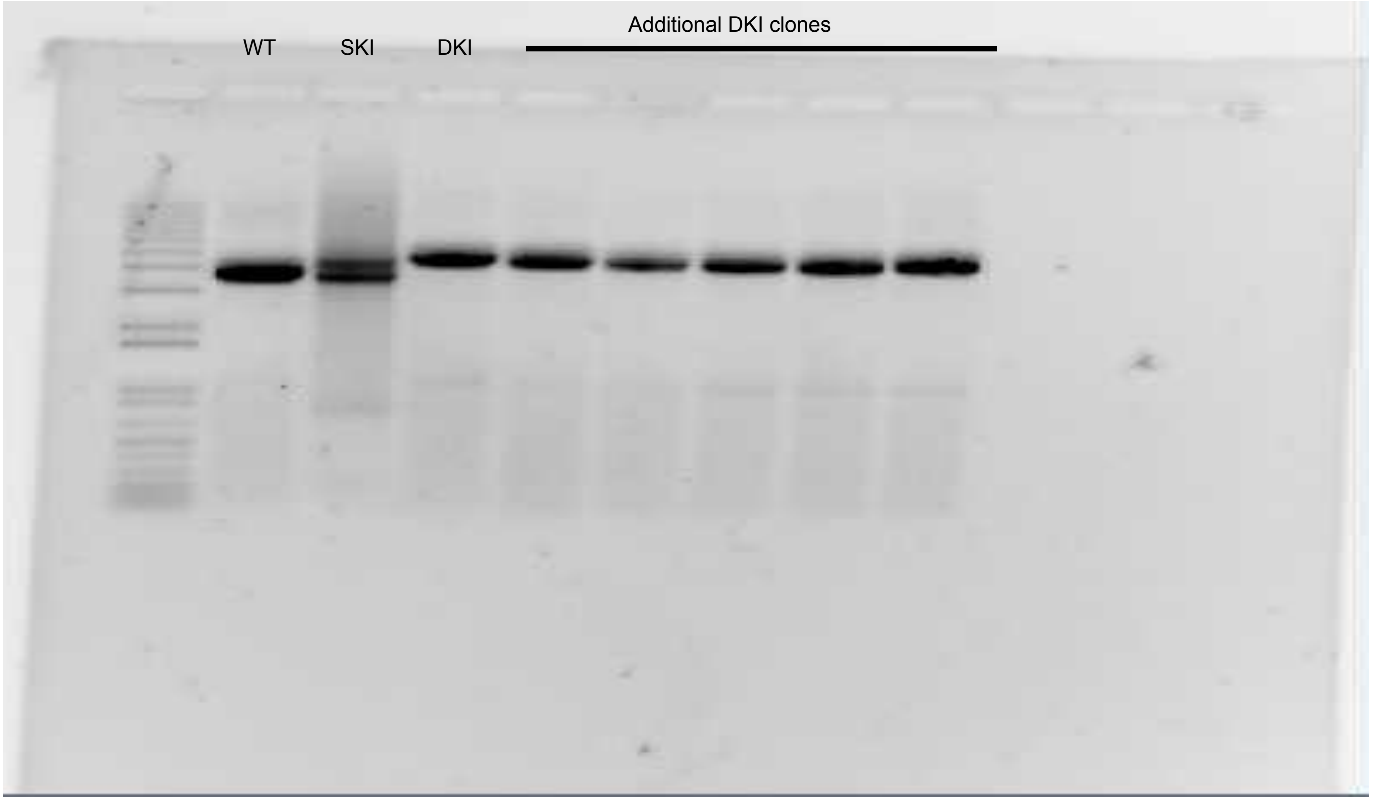

Supplementary Figure S7. PCR amplification of the LC1 locus. Full-length gel from which Fig. 1b was cropped. First 3 lanes show amplification of the LC1 locus from parental cells (WT), single knock-in cells (SKI), and double knock-in cells (DKI) as shown in Fig. 1b. Additional lanes show additional clones of DKI.

Supplementary Video Legends

Supplementary Video S1. Live video microscopy of parasites in whole blood from an infected mouse. Fluorescence microscopy of undiluted blood from a mouse infected with wild-type *T. brucei* parasites expressing mCherry. Bright-field illumination is turned on at time stamp 0:07 (upper left), then dimmed and raised again to show the density of red blood cells. Given the thickness of the chambers (70 -100 µm), individual red blood cells are not distinguishable. Supplementary Videos S1, S4, and S5 are from three independent mice.

Supplementary Video S2. Fluorescence microscopy of DKI parasites in whole blood from an infected mouse. Live video microscopy of undiluted blood from a mouse infected with DKI parasites expressing mCherry. Parasites have a beating flagellum, but do not translocate. Supplementary Videos S2 and S3 are from independent mice.

Supplementary Video S3. Fluorescence microscopy of DKI parasites in whole blood from an infected mouse. Live video microscopy of undiluted blood from a mouse infected with DKI parasites expressing mCherry. Parasites have a beating flagellum, but do not translocate. Supplementary Videos S2 and S3 are from independent mice.

Supplementary Video S4. Fluorescence microscopy of WT parasites in whole blood from an infected mouse. Live video microscopy of undiluted blood from a mouse infected with WT parasites expressing mCherry. The anterior (thin) and posterior (thick) ends of the cells can be distinguished, with the

anterior also showing more rapid movements. Most cells oscillate back and forth remaining close to the point of origin, while a few exhibit short periods of translocation interrupted with pauses and reversals. Circle highlights a cell that undergoes translocation for a few seconds with the posterior end leading before switching direction as indicated by the arrow at time stamp 0:09 (upper left). Supplementary Videos S1, S4, and S5 are from three independent mice.

Supplementary Video S5. Fluorescence microscopy of WT parasites in whole blood from an infected mouse. Live video microscopy of undiluted blood from a mouse infected with WT parasites expressing mCherry. The anterior (thin) and posterior (thick) ends of the cells can be distinguished, with the anterior also showing more rapid movements. Most cells oscillate back and forth remaining close to the point of origin, while a few exhibit short periods of translocation interrupted with pauses and reversals. Top left circle highlights a cell that undergoes saltatory translocation with the anterior end leading. Bottom right circle highlights a cell that undergoes saltatory translocation with the posterior end leading. Supplementary Videos S1, S4, and S5 are from three independent mice.
